# Analysis of Circulating Vascular Endothelial Growth Factor and Its Soluble Receptors in Patients with Different Forms of Chronic Urticaria

**DOI:** 10.1155/2015/578383

**Published:** 2015-02-10

**Authors:** Julia Jagodzinska, Renata Polaniak, Ewa Birkner, Alicja Kasperska-Zajac

**Affiliations:** ^1^Anaesthesiology and Intensive Therapy, Upper Silesian Medical Centre, 40-635 Katowice, Poland; ^2^Department of Nutrition-Associated Disease Prevention, Faculty of Public Health, Ulica Piekarska 18, Medical University of Silesia in Katowice, 41-902 Bytom, Poland; ^3^Department of Biochemistry, Ulica Jordana 19, Medical University of Silesia in Katowice, 41-808 Zabrze, Poland; ^4^Chair and Clinical Department of Internal Diseases, Dermatology and Allergology, Ulica M. Curie-Skłodowskiej 10, Medical University of Silesia in Katowice, 41-800 Zabrze, Poland

## Abstract

*Background*. Vascular endothelial growth factor (VEGF) is a powerful enhancer of vascular permeability and inflammatory response; however its significance in chronic urticaria is poorly recognised. *Aim*. To compare free circulating levels of VEGF and its soluble receptors (sVEGFR1 and VEGFR2) in patients with different forms of chronic urticaria. *Methods*. The concentrations of VEGF and its receptors in plateletpoor plasma (PPP)/plasma were measured using enzyme-linked immunosorbent assay in chronic urticaria: (1) chronic spontaneous urticaria (CSU) with positive autologous serum skin test (ASST), (2) CSU with negative response to ASST, (3) CSU with concomitant euthyroid Hashimoto's thyroiditis (CSU/Hashimoto), (4) delayed pressure urticaria (DPU), and the healthy subjects. *Results*. There were no significant differences in VEGF concentration in PPP between CSU groups and the healthy subjects. Contrary, VEGF concentration was significantly higher in DPU and CSU/Hashimoto patients as compared with the healthy subjects and CSU groups. Furthermore, VEGF value in CSU/Hashimoto patients during the remission was similar to that of the active period and significantly higher than the healthy subjects; VEGF concentration was significantly correlated with TSH. Plasma concentrations of sVEGF1 and sVEGF2 were similar in chronic urticaria patients and the healthy subjects. *Conclusions*. Increased free circulating VEGF concentration may result from the urticarial process itself as well as concomitant Hashimoto's thyroiditis.

## 1. Introduction

Chronic spontaneous urticaria (CSU) is characterised by basophiles/mast cells activation accompanied by systemic inflammatory response and neuroimmunendocrine dysfunction and coagulation/fibrinolysis activation [1–5]. An interplay between hemostasis and inflammation has been proposed as a pathomechanism in urticaria. Whether coagulation/fibrinolysis activation has a primary role in the pathogenesis of the disease or simply acts as an amplification system should be defined [2, 6–8].

Urticarial wheals/angioedema result from vascular dilatation and leakage of fluid into the skin in response to histamine and other mediators released from different cells. Histamine is believed to be a major mediator in urticarial lesions; however it is well known that antihistamine therapy is ineffective in many patients, suggesting that there may be other contributing factors [9]. It has been suggested that vascular endothelial growth factor (VEGF) plays a role in the pathogenesis of chronic urticaria [10]. VEGF is a powerful enhancer of vascular permeability, being 50,000 times more potent than histamine [11]. In addition, VEGF is released by mast cells and other cells associated with chronic urticaria and stimulates mast cell migration [12], and its synthesis is induced by histamine [13].

Only limited data exist describing the association of VEGF with urticaria. Thus, the aims of the present study were 3-fold: (i) to compare circulating levels of VEGF using platelet poor plasma (PPP) of patients with different forms of chronic urticaria: (I) CSU with negative response to autologous serum skin test (ASST), (II) chronic autoreactive urticaria (with positive response to ASST) with or without coexistent untreated euthyroid Hashimoto's thyroiditis, and (III) delayed pressure urticaria (DPU) and appropriate controls, (ii) to compare circulating levels of VEGF between active period of the disease and the clinical remission phase, and (iii) to assess circulating levels of soluble receptors of VEGF (sVEGFR1 and VEGFR2) in plasma of the patients with chronic urticaria.

## 2. Material and Methods

### 2.1. Subjects

Ninety-one patients with active chronic urticaria were enrolled in the study. Their clinical characteristics are shown in Table 1.

The activity/severity of CSU was assessed according to the urticaria activity score (UAS) system, estimated during two days. Briefly, the number of the wheals and severity of pruritus were scored as follows: number of wheals, 0 = no wheals, 1 = mild wheals (<20 wheals/24 h), 2 = moderate wheals (21–50 wheals/24 h), and 3 = intense wheals (>50 wheals/24 h, or large confluent areas of wheals); pruritus, 0 = none, 1 = mild, 2 = moderate, and 3 = intense. Our patients' scoring was from 3 to 6.

ASST and other investigations had been performed to exclude any known causes of the diseases or the concomitant diseases. Among those, routine dental and laryngological consultations were performed to exclude the infectious foci.

None of the examined subjects had taken oral corticosteroids or antidepressants within 8 weeks or antihistamines within at least 4 days before the study.

Chronic urticaria patients were divided into four groups.

- *CSU/ASST(−)*. 40 patients with negative response to ASST suffering from CSU were enrolled.
- *CSU/ASST(+)*. 26 patients with positive response to ASST suffering from CSU-autoreactive urticaria were enrolled. All other identified causes of the diseases had been excluded.
- *DPU*. 9 patients with pure DPU (DPU alone) and three patients with concomitant CSU were enrolled into the study. All identified causes of CSU, including autoreactivity and concomitant diseases, had been excluded.
- *CSU/ASST(+)/Hashimoto Group*. 13 patients with positive response to ASST suffering from CSU and coexistent untreated euthyroid Hashimoto's thyroiditis were enrolled. The patients were examined twice: first, during the active period, and next, during the clinical remission. Seven patients who had previously showed active skin lesions were classified for the second stage of the study performed when the clinical remission was reached. All patients were in spontaneous remission and had been medication-free for at least 3 weeks—*CSU/ASST(+)/Hashimoto Subgroup in Remission*. The remaining 6 patients, participating in the previous stage, failed to reach the spontaneous remission phase.

Euthyroid Hashimoto's thyroiditis diagnosis was based upon coexistence of sonographic (US) changes of the thyroid and abnormally high thyroid peroxidase antibodies titers (anti-TPO).

*Biochemical parameters and thyroid echogenicity of CSU/ASST(+)/Hashimoto group:*

- thyroid-stimulating hormone (TSH): (median: 3.5; range: 2.75–4.0 *μ*IU/mL),
- free thyroxine (FT4): (median: 1.11; range: 0.95–1.4 ng/dL),
- anti-TPO: (median: 313; range: 220–535 IU/mL),
- thyroid US abnormalities.

The control group consisted of 34 nonatopic, nonsmoking healthy subjects (10 males, 24 females; aged 18–45 years; median 38 years).

The Ethics Committee of the Medical University of Silesia approved of the study (NN-6501-171/07) and written, informed consent was obtained from all the subjects participating.

### 2.2. Blood Samples and Analytical Methods

Because platelets are potential source of VEGF, its concentration was measured in platelet poor plasma (PPP). Blood was obtained in the morning (7.00 a.m. to 8.00 a.m.; in the fasting state) after a 25-minute rest at slight or no stasis from the antecubital vein into CTAD tubes, which contain four anticoagulants, sodium citrate, theophylline, adenosine, and dipyridamole (Vacutainers, Becton-Dickinson), to obtain maximal stabilization of platelets, and then placed into the ice/water bath. The tubes were then centrifuged at 3000 g for 15 minutes at 4°C. Following the first centrifugal cycle 3/4 of the top plasma was removed with a plastic transfer pipette. This plasma was centrifuged again at 3000 g for 15 minutes, to remove the residual platelets. The plasma obtained was stored at −70°C until assayed for VEGF.

sVEGF-R1 and sVEGF-R2 concentrations were performed in the plasma collected, using EDTA as an anticoagulant.

### 2.3. VEGF Analysis

VEGF concentration was determined using enzyme-linked immunosorbent assay (ELISA; R&D Systems Inc., Minneapolis, MN, USA), to detect the isoforms (VEGF121 and VEGF165). The detection limits were 9.0 pg/mL. Values below 9 pg/mL were equalized to zero.

### 2.4. sVEGF-R1 and sVEGF-R2 Analyses

The cytokines plasma concentrations were assayed by specific, commercially available, ELISA assay kits (R&D Systems Inc., Minneapolis, MN, USA), in accordance with the manufacturer's instructions. The sensitivity of the assay for VEGF-R1 and sVEGFR-2 was 3.0 pg/mL and 5 pg/mL, respectively.

### 2.5. Other Laboratory Investigations

Serum concentrations of TSH, fT4, and anti-TPO were measured using an electrochemiluminescence immunoassay (ECLIA) method (Roche Diagnostics GmbH, Mannheim, Germany) with a detection limit of 0.005 *μ*IU/mL, 0.023 ng/dL, and 5 IU/mL, respectively. Normal lab ranges were TSH [0.27–4.2] *μ*IU/mL; fT4 [0.93–1.7] ng/dL; anti-TPO [0–34] IU/mL.

The blood platelet and leukocytes counts were determined using an automatic haematology analyser.

### 2.6. Autologous Serum Skin Test (ASST)

Intradermal tests with patient's own serum were performed according to the method described by Sabroe et al. [14]. A serum-induced wheal of diameter greater by at least 1.5 mm than that of a control wheal induced with physiological saline was accepted as positive.

### 2.7. Statistical Analysis

#### 2.7.1. Data Were Delivered as Medians and Interquartile Range (IQR)

The Mann-Whitney *U* test was used to compare results from CSU group and the controls. The Kruskal-Wallis variance analysis was used to screen differences between CU groups. The Wilcoxon's paired test was employed to compare the CSU/ASST(+)/Hashimoto subgroups. Correlation coefficient was obtained by Spearman test. *P* values lower than 0.05 were considered significant.

## 3. Results

### 3.1. VEGF Concentration in PPP

**Figure 1:**
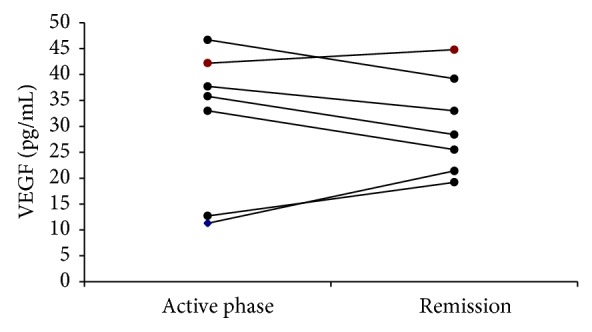
Changes in plasma VEGF concentration in CSU/ASST(+)/Hashimoto patients during the active phase in comparison to the remission phase (*P* = 0.656).

There were no significant differences in VEGF concentration between CSU/ASST(−), CSU/ASST(+), and the healthy subjects (Table 2). Contrary, VEGF concentration was significantly higher in DPU and CSU/ASST(+)/Hashimoto groups as compared with the healthy subjects and CSU groups (Table 2). Furthermore, VEGF value in PPP of CSU/ASST(+)/Hashimoto subgroup during the remission period was similar to that of the active period (median: 28.4 versus 35.8; *P* > 0.05; Figure 1) and significantly higher than the healthy subjects (median: 28.4 versus 18.4 pg/mL; *P* < 0.05).

### 3.2. Plasma sVEGF-R1 and sVEGF-R2 Concentrations

There were no significant differences in the receptors concentration between all investigated groups (Table 2).

### 3.3. Correlations

There were no significant correlations between VEGF, sVEGF-R1, sVEGF-R2, and platelet counts in chronic urticaria and the healthy subjects (Table 3). PPP/plasma VEGF, sVEGF-R1, and sVEGF-R2 concentrations were not significantly correlated with the disease activity as assessed by UAS (data not shown). Plasma VEGF concentration was significantly correlated with TSH, but not with anti-TPO levels (*r* = 0.63, *P* = 0.02; *r* = 0.123, *P* = 0.767, resp.) in CSU/ASST(+)/Hashimoto patients. There were no significant correlations between VEGF concentration and count of WBC and all five subtypes of the cells (Table 4).

## 4. Discussion

VEGF concentrations were measured in PPP, which seems the best way to assess* in vivo* free circulating VEGF, because platelets are an important source of VEGF in the circulation [15].

It is known that the activation of coagulation/fibrinolysis parallels the activity of CSU [6]. However, the significance of systemic platelet activity in CSU is unclear [16, 17].

In our study VEGF concentrations were significantly higher in DPU patients and patients with autoreactive CSU with coexistent untreated euthyroid Hashimoto's thyroiditis as compared with the healthy subjects. In addition, plasma VEGF concentrations in CSU/ASST(−) and CSU/ASST(+) groups were not statistically different from those observed in the normal controls.

Contrary to our results, Tedeschi et al. found the overproduction of VEGF in CSU significant, including the increased immunohistochemical expression in skin and plasma concentration. It has been suggested that VEGF may play an important role in the increased vascular permeability, oedema, and inflammatory infiltrate [10]. It is possible that several points may explain this discrepancy. There are some differences in samples examined (PPP versus plasma). In addition, this discrepancy might be explained by the differences in CSU severity/activity between the studies [18].

In contrast to chronic urticaria, VEGF was not dramatically upregulated in two cases of acute urticaria; only weak expression was detected in rare mononuclear inflammatory cells [19]. It is possible that more intensive expression of VEGF would be found in cases of more extensive inflammatory changes. In addition, VEGF levels were not elevated in skin or in plasma of patients with an increased number of mast cells, such as mastocytosis [20]. Contrary, overproduction of VEGF has been observed in bullous disorders [19] and atopic dermatitis [21].

In our study patients with autoreactive chronic urticaria with coexistent euthyroid Hashimoto's thyroiditis (CSU/ASST(+)/Hashimoto group) were examined twice: first, during the active period, and next, during the clinical spontaneous remission. We found similar plasma VEGF concentration in CSU/ASST(+)/Hashimoto group during the two periods. In addition, these values were significantly higher from those of the healthy subjects. Significant association was found between concentrations of VEGF and TSH in the patients. It seems that overproduction of VEGF is a persistent phenomenon associated with untreated Hashimoto's thyroiditis resulting from higher TSH level, rather than CSU itself. However, the sample size is too small for solid conclusions.

These results may confirm previous observation, suggesting that VEGF may be one of the important thyroid angiogenic factors responsible for goiter formation [22], probably produced by thyroid follicles in response to stimulators of TSH receptors [23]. There was a close relationship between serum VEGF and TSH levels in patients with Graves' disease or Hashimoto's thyroiditis [22]. In addition, serum VEGF concentration was significantly higher in patients with untreated hypothyroid goitrous Hashimoto's thyroiditis and was reduced after therapy [22].

Plasma VEGF concentration was increased in patients with DPU. The major sources of VEGF in these patients remain speculative. This increase might be produced by VEGF released from leukocytes and platelets into the circulation or by exudation of the cytokine into the blood stream from inflamed skin.

VEGF may be released by mast cells and inflammatory cells involved in the local urticarial response in DPU [24–26]. It has been indicated that eosinophils are the main cellular source of VEGF in CSU lesional skin [10]. In light of previous [10] and current observations, it is possible that VEGF facilitates vascular permeability and activation of inflammatory cells leading to amplification of the inflammatory reaction in DPU.

It has been indicated that VEGF in the bloodstream is transported, predominantly by platelets and neutrophils (approximately 60% in neutrophils and 34% in platelets) [27]. It seems that the sources of VEGF in DPU may not directly relate to mast cells and local inflammatory cells but rather to the circulating cells. Taking into account that platelets derived chemokines may be released in DPU [28], but not in CSU [29], it seems that these cells may contribute to increased concentration of VEGF in DPU. However, no significant association was found between platelet and neutrophil counts and VEGF concentration, probably due to the small sample size.

There were no correlations between concentrations of VEGF and its soluble receptors (sVEGFR1 and sVEGFR2) in chronic urticaria patients.

## 5. Conclusions

Increased free circulating VEGF concentration may result from the urticarial process itself as well as concomitant Hashimoto's thyroiditis.

The hypothesis that local or systemic overproduction of soluble VEGF plays a role in the pathogenesis of chronic urticaria should be subject of further studies.

## Figures and Tables

**Table 1:** Clinical characteristic of chronic urticaria patients.

Parameters	CSU/ASST(−)	CSU/ASST(+)	CSU/ASST(+)/Hashimoto	DPU
*n*	40	26	13	12
Sex (F/M)	28/12	18/8	13/0	5/7

Age	38	37	36	40
(Range; years)	(32–45)	(27–45)	(28–42)	(37–45)

Disease duration	11.5	10	10	21
(Range; months)	(3–48)	(6–35)	(4–25)	(8–52)

Concomitant angioedema	4	4	2	—

*n*: number of patients; CSU: chronic spontaneous urticaria; DPU: delayed pressure urticaria; ASST: autologous serum skin test; data are presented as median and range.

**Table 2:** Plasma concentration of VEGF, VEGF-R1, and VEGF-R2 and number of platelets in chronic urticaria patients and healthy subjects.

Analysed parameters (unit)	Chronic urticaria				Control (*n* = 34)
	CSU/ASST(−) (*n* = 40)	CSU/ASST(+) (*n* = 26)	CSU/ASST(+)/ Hashimoto (*n* = 13)	DPU (*n* = 12)	
	Median (IQR)	Median (IQR)	Median (IQR)	Median (IQR)	Median (IQR)
VEGF (pg/mL)	19.80 (7.65–27.05)	23.35 (9.8–26.8)	*P* = 0.0003 37.7 (33.45–42.55)	*P* = 0.0118 29.85 (21.3–41.5)	18.3 (0–24.5)

sVEGF-R1 (pg/mL)	36.50 (27.35–46)	41.00 (34.1–46.80)	47.50 (36.225–50.45)	32.05 (27.05–42.25)	39.40 (26.6–24.2)

sVEGF-R2 (pg/mL)	7998 (6335–10215)	8670 (5980–11980)	9033 (7491.75–11964.5)	10076 (6109–12973.5)	7536.5 (5479–9295)

Platelets (×10^9^/L)	240 (192–316)	226 (187.0–267)	217 (186–254.75)	230 (212.5–265.5)	230.5 (190–251)

*n*: number of patients; CSU: chronic spontaneous urticaria; DPU: delayed pressure urticaria; ASST: autologous serum skin test; IQR: interquartile range; *P*: statistically significant values as compared with the control group.

**Table 3:** Correlations between VEGF, sVEGF-R1, sVEGF-R2, and platelet number in chronic urticaria and healthy subjects.

Spearman test	Chronic urticaria				Healthy subjects (*n* = 34)
	CSU/ASST(−) (*n* = 40)	CSU/ASST(+) (*n* = 26)	CSU/ASST(+)/Hashimoto (*n* = 13)	DPU (*n* = 12)	
VEGF/sVEGF-R1					
*P*	0.653	0.905	0.764	0.905	0.930
*r*	−0.073	0.024	−0.093	0.038	0.015
VEGF/sVEGF-R2					
*P*	0.929	0.440	0.877	0.528	0.533
*r*	0.014	−0.296	−0.049	−0.202	−0.110
sVEGF-R1/sVEGF-R2					
*P*	0.093	0.681	0.778	0.896	0.136
*r*	0.268	0.084	0.087	−0.042	0.260
VEGF/platelet					
*P*	0.782	0.735	0.820	0.430	0.892
*r*	0.045	0.069	−0.071	−0.251	0.024

**Table 4:** Correlations between plasma VEGF concentration and count of WBC and all five subtypes of the cells in delayed pressure urticaria (DPU).

Spearman test	DPU (*n* = 12)
WBC	
*P*	0.125
*r*	−0.491
Neutrophils	
*P*	0.465
*r*	0.124
Eosinophils	
*P*	0.627
*r*	−0.051
Basophils	
*P*	0.745
*r*	0.278
Lymphocytes	
*P*	0.243
*r*	0.569
Monocytes	
*P*	0.744
*r*	0.132

WBC: white blood cell count.

## Abbreviations

CU: Chronic urticaria
CSU: Chronic spontaneous urticaria
DPU: Delayed pressure urticaria
ASST: Autologous serum skin test
UAS: Urticaria activity score
VEGF: Vascular endothelial growth factor
PPP: Platelet poor plasma
TSH: Thyroid-stimulating hormone
sVEGFR1 and VEGFR2: Soluble receptors of VEGF.
